# Introduction to Epitaxial growth of nanostructures and their properties

**DOI:** 10.1039/d3na90054a

**Published:** 2023-06-02

**Authors:** Jin Zou

**Affiliations:** a University of Queensland Australia

## Abstract

Professor Jin Zou introduces the *Nanoscale Advances* themed collection on Epitaxial growth of nanostructures and their properties.
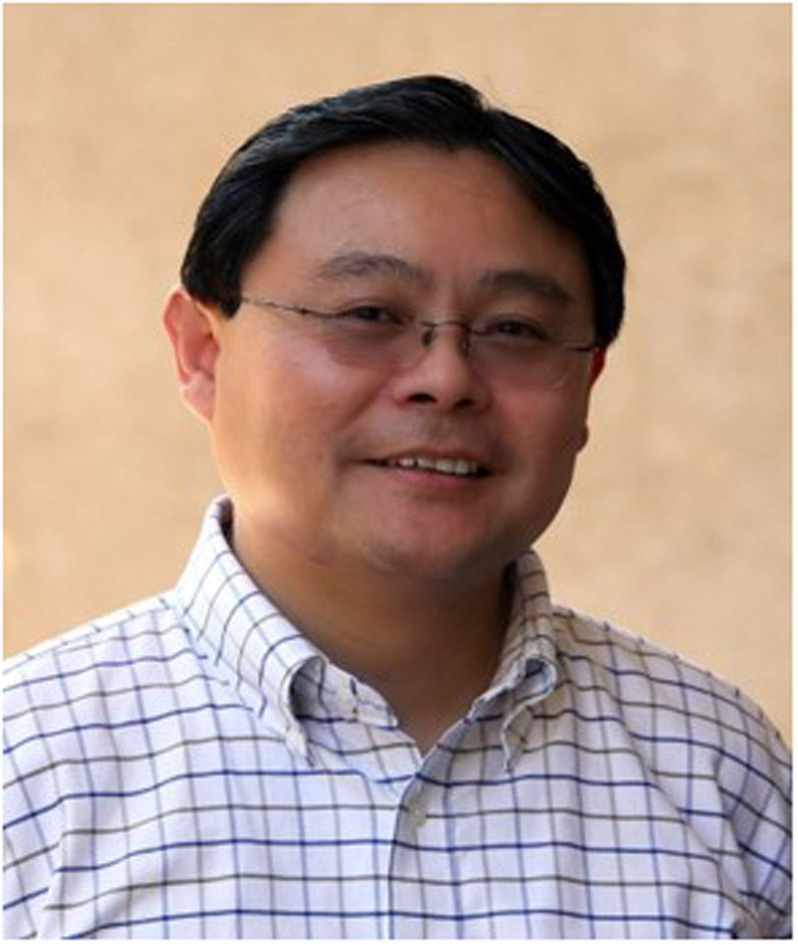

Nanoscience and technology have been identified as a key discipline of science and technology that manipulates materials at the nanoscale, from which unusual physical, chemical, and biological properties can emerge in the nanostructured materials. These properties often differ from the properties of their bulk counterparts, thus generating significant breakthroughs in nanomaterial design and manufacturing.

To realise the manufacturing of nanoscale systems, advance deposition techniques are often used, in which the epitaxial growth of nanomaterials has been widely employed to grow a wide array of new nanostructures that provide unprecedented opportunities in developing new nanomaterial systems with unique physiochemical properties and vast application potentials.

Epitaxial growth of nanostructures refers to the nanostructure growth process, in which crystalline nanostructures are grown on a substrate surface with certain crystallographic relationships between the nanostructures and their underlying substrates. This process has been widely employed to produce nanoscale structures with precise control over the morphology, structure, and chemistry of the nanostructures, as well as their orientations. The nanostructures, grown under such crystallographically rigid and morphologically and chemically flexible conditions, often exhibit unique properties, making them attractive for a huge range of applications in fields such as electronics, energy, and biomedical engineering.

In this themed collection, 3 review and 7 research articles are presented in the areas of semiconductor nanostructures (such as quantum dots and semiconductor nanowires), 2D nanostructures (including ultra-thin nanosheets), and hierarchical nanostructured metal–organic frameworks (MOF-on-MOF structures).

Epitaxial heterostructure design for electrochemical applications is reviewed, covering synthesis strategies for creating epitaxial interfaces and the superiorities of epitaxial heterostructures in electrocatalysis, supercapacitors and batteries (https://doi.org/10.1039/D2NA00710J). Additionally, epitaxial growth of quantum dots during III–V nanowire growth is reviewed, discussing how quantum dots can be manipulated and the growth controlled (https://doi.org/10.1039/D2NA00956K). Furthermore, epitaxial growth of GaN-based nanowire heterostructures on various substrates and their applications in piezoelectric nanogenerators, light-emitting diodes, and solar-driven water splitting are reviewed (https://doi.org/10.1039/D2NA00711H).

Research works are presented such as the achievement of epitaxial growth of long GaAs–AlGaAs nanowire heterostructures on a wafer-scale Si substrate through a self-catalysing approach (https://doi.org/10.1039/D2NA00848C). The system demonstrates bright and homogeneous photoluminescence, enhanced by the presence of GaAs–AlGaAs core–shell heterostructures. Elsewhere, the nucleation and growth of epitaxial GaN nanowires on sapphire substrates are systematically investigated to understand the impact of growth parameters on the nanowire qualities (https://doi.org/10.1039/D2NA00939K). Fluorescence enhancement of semiconductor nanowire heterostructures is also investigated using Al_2_O_3_–GaP core–shell nanowire heterostructures, from which excitation enhancement has been achieved by manipulating its wavelength (https://doi.org/10.1039/D2NA00749E). Synthetic methods are also presented, including formation of epitaxy in silicene (2D silicon) on an Ag (111) substrate driven by 2D solid-phase crystallization of deposited amorphous Si on Ag (111) through post-growth annealing, in which using an e-beam to selectively write ‘Si pixels’ has been demonstrated (https://doi.org/10.1039/D2NA00546H). Epitaxial growth of high-quality chromium chalcogenide nanoplates on mica is carefully studied by investigating the interfaces of chromium chalcogenides and mica during nanostructure nucleation and growth at the atomic level (https://doi.org/10.1039/D2NA00835A). The impact of steric hindrance at the interface of non-isostructural MOF-on-MOF systems is also investigated (https://doi.org/10.1039/D2NA00790H). Finally, a demonstration of polarization-dependent plasmonic heating in multilayered MOF thin films with preferred orientation is presented with embedded Ag nanoparticles, showing the anisotropic optical functionality in MOF thin films that enables applications such as efficient reactivation and partial catalytic reactions, as well as soft nanorobotics (https://doi.org/10.1039/D2NA00882C).

## Supplementary Material

